# Evaluation of Iris Melanoma with Anterior Segment Optical Coherence Tomography

**DOI:** 10.4274/tjo.66742

**Published:** 2017-08-15

**Authors:** Mehtap Arslantürk Eren, Ahmet Kaan Gündüz, Özlenen Ömür Gündüz

**Affiliations:** 1 Ankara Üniversitesi Faculty of Medicine, Department of Ophthalmology, Ankara, Turkey

**Keywords:** Iris, melanoma, optical, coherence, tomography

## Abstract

Anterior segment optical coherence tomography (AS-OCT) is a relatively new imaging modality that allows assessment of anterior segment structures. AS-OCT enables the differentiation of benign and malignant tumors through the evaluation of lesion size, internal structure, degree of vascularity, and anterior and posterior surfaces. Herein, we discuss the AS-OCT findings of a patient with spindle type iridociliary melanoma diagnosed in pathologic examination.

## INTRODUCTION

Uveal melanomas are the most common primary intraocular malignancy in adults, and iris melanomas account for 3-10% of uveal melanomas.^1^ Iris melanoma, the most common malignancy of the iris, originates from the melanocytes of the iris stroma and its prognosis is better than that of choroid and ciliary body melanomas. With the examination techniques currently available, the diagnosis of uveal melanomas has reached 99%.^2^ Uveal melanoma differs in this respect from other systemic cancers, for which biopsy is the diagnostic gold standard. Slit-lamp examination, transillumination, digital photography, A-scan ultrasonography, B-scan ultrasonography (USB), ultrasonic biomicroscopy (UBM), fluorescein and indocyanine green angiography, and anterior segment optical coherence tomography (AS-OCT) are methods utilized in diagnosis.

Using anterior segment imaging methods to visualize lesion features such as location and thickness and to determine whether lesions are solid or cystic, limited to the iris or involve the ciliary body are important for diagnosis and treatment planning in patients with iris or iridociliary lesions. One of these methods, AS-OCT, enables the acquisition of cross-sectional images of the tissues with low coherence interferometry.

Evaluation of the anterior segment with OCT was first performed in 1994 by Izatt et al.^3^ AS-OCT (developed from retinal OCT, which acquires images using 830 nm wavelength light^3^) is a noncontact method that provides high-resolution anterior segment images using 1310 nm wavelength light.^4^ In this report, we share a case in which an iris mass was detected in routine eye examination, partial lamellar sclerouvectomy (PLSU) was planned following AS-OCT imaging, and a diagnosis of malignant melanoma was histopathologically confirmed.

## CASE REPORT

**Figure 1:**
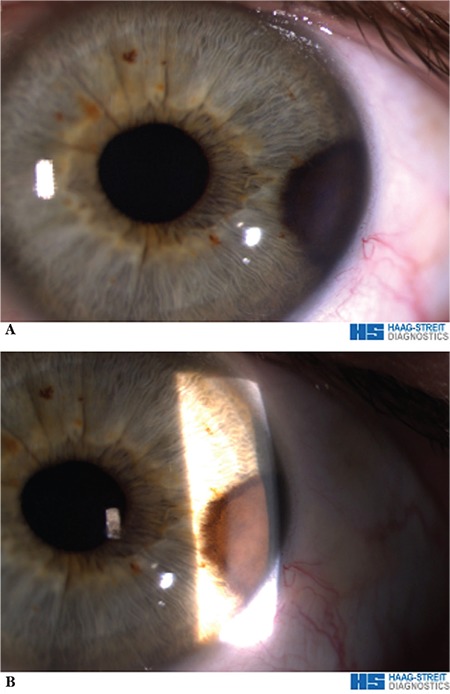
A) A raised brown mass extending to the iris root is visible on the peripheral iris surface between the 14:30-15:30 clock hours. B) Biomicroscopic view of the mass

A 56-year-old female patient presented to our clinic with complaints of decreased vision in both eyes for the past several months. Her best visual acuity was 3/10 in the right eye and 4/10 in the left eye. Biomicroscopic examination revealed no pathology other than nuclear cataract in the right eye. In the iris of the left eye, a hyperpigmented mass approximately 4x2.5x1.5 mm in size was observed situated between 14:30-15:30 clock hours and extending to the iris root and anterior chamber (Figure 1).

Secondary cataract was present in the left eye; intraocular pressure measured by Goldmann applanation tonometer was 13 mmHg in both eyes and fundus examination was normal in both eyes. Further inquiry into the patient’s history revealed that she had been aware of the iris spot since childhood but had never consulted an institution or doctor because she did not consider it important. There was nothing remarkable in her personal or family histories.

**Figure 3:**
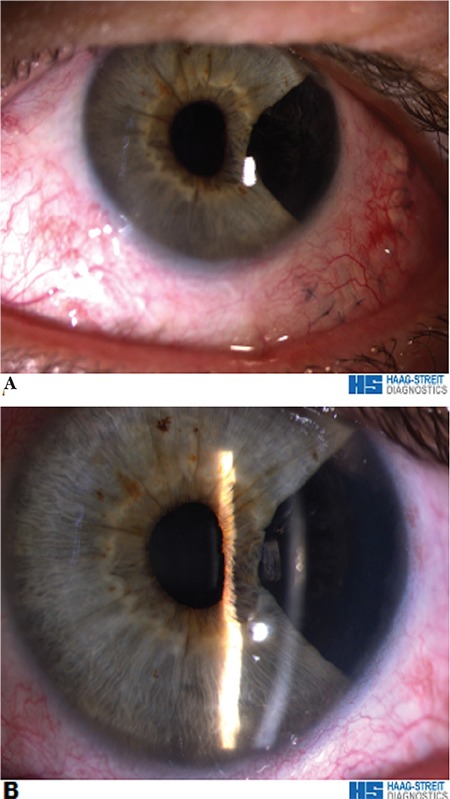
Postoperative anterior segment photograph of the patient. A) Iris coloboma developed postoperatively. B) Biomicroscopy image

**Figure 4:**
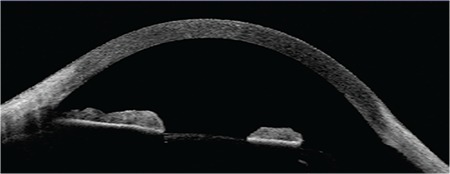
The iris periphery does not appear on postoperative anterior segment optical coherence tomography due to the surgery (enhanced anterior segment single). There is no residual mass

AS-OCT (Visante OCT/Zeiss) examination revealed that the mass was 2.30x1.32 mm in size, was raised with distinct borders and a smooth anterior surface, was solid and heterogeneous (with a vascular component), and extended to the ciliary body. While the anterior surface of the mass was defined by high reflectivity, the posterior surface boundaries could not be distinguished (Figure 2A, 2B). Based on the biomicroscopy and AS-OCT findings, a prediagnosis of malignant melanoma was made and iridogoniocyclectomy through PLSU was planned. Under hypotensive general anesthesia, surgery was placing the patient in reverse Trendelenburg position. Intraoperative transillumination to determine the location of the tumor showed that it did not extend beyond the pars plana region, and iridogoniocyclectomy was performed via PLSU. The tumor was excised with wide surgical margins and was sent to pathology. There were no intraoperative or postoperative complications. Postoperative examination revealed no residual mass (Figures 3, 4). Histopathological diagnosis was reported as mixed spindle A and B type melanoma. The patient was followed without additional treatment and no recurrence was detected during 23 months of follow-up. Phacoemulsification with posterior chamber intraocular lens implantation was performed on the left eye in the postoperative 12^th^ month due to cataract. After 22 months of follow-up, the patient later developed rhegmatogenous retinal detachment and underwent a pars plana vitrectomy with silicone tamponade. The patient was in stable clinical condition at 1-month follow-up after vitreoretinal surgery.

## DISCUSSION

In iris melanomas, the most common symptoms at admission are a blotch or color change in the iris. Iris melanomas can vary in appearance, ranging from amelanotic to brown, and are usually located in the lower half of the iris. They usually grow locally on the surface of the iris or toward the anterior chamber, though they can also extend toward the anterior chamber angle or the ciliary body.

Findings that suggest iris melanoma include vascularization of a mass on the surface of the iris, retraction of the pupil toward the lesion, a mass surface that is uneven and nodular instead of homogenous, invasion of the iridocorneal angle, and pigment covering the trabecular meshwork.^5^

Transillumination, anterior segment photographs, USB, UBM, AS-OCT, and fluorescein and indocyanine green angiography are utilized to visualize the anterior segment. UBM provides valuable information about the anterior segment because it has high penetration strength, can demonstrate extension to the ciliary body, is unaffected by pigmentation, and allows clear imaging of a tumor’s posterior margin. Marigo et al.^6^ used UBM to determine lesion size, internal structure, and extension toward the ciliary body or surrounding tissues and compared these findings with the lesions’ histopathologic appearance after excision. They found that there was similarity between UBM findings and histopathological findings.

AS-OCT is an imaging technique used in different areas of ophthalmology such as cornea, refractive surgery, glaucoma, and ocular tumors, and its use has steadily increased over the last 10-20 years. AS-OCT provides high-resolution cross-sectional images without contact with the eye.^7^

The instrument can acquire a 256 A-Scan low-resolution image in 125 ms or 512 A-Scan high-resolution image in 250 ms. Thus, the resolution can be approximately 18 μm axial with 3-6 mm depth of penetration, and it is used in the imaging of many pathological conditions such as iris cysts, iris nevi, and iris/ciliary body melanomas. There are many studies comparing AS-OCT with other anterior segment imaging methods. Pavlin et al.^8^ demonstrated that AS-OCT is useful in small hypopigmented tumors limited to the iris but that UBM is superior to AS-OCT in imaging highly pigmented and ciliary body tumors due to its high tissue penetration.

Hau et al.^9^ compared USB with AS-OCT in the examination of 126 eyes with iris or iridociliary body lesions. They demonstrated that the axial resolution was higher in AS-OCT compared to USB and that AS-OCT was superior in visualizing lesions involving the lateral and anterior aspects of the iris.

In contrast, they demonstrated that USB is superior in showing tumor configuration and internal structure in pigmented iris melanomas or lesions extending posteriorly because sound waves provide better penetration into pigmented lesions than light energy.

In AS-OCT images, reflectivity is correlated with the degree of tissue pigmentation. In a normal iris, the stroma is moderately reflective and the anterior surface is highly reflective. The iris pigment epithelium forms a highly reflective border on the posterior surface of the iris. Melanotic lesions show greater reflectivity on AS-OCT, while the reflectivity of amelanotic lesions is equal to or lower than that of the stroma. In one study, it was observed that iris nevi show high reflectivity, while iris melanomas contained areas of varying degrees of reflectivity dispersed throughout the thickness of the mass.^10^ In the same study, it was reported that AS-OCT, especially in high-resolution mode, provided information comparable to UBM about lesion location, internal structure, and extension to the anterior chamber. Iris nevi and melanomas show low-to-moderate reflectivity on UBM, independent of the degree of pigmentation.^11^

In our case, the base diameter of the lesion was measured as 2.30 mm and its thickness as 1.32 mm with anterior segment OCT. The internal structure of the lesion appeared heterogeneous due to the vascular component and the posterior border of the pigmented lesion was not distinguishable. It has been stated in earlier studies that a melanotic lesion of the iris that has a diameter greater than 3 mm and a thickness greater than 1 mm or shows documented growth during follow-up may be interpreted as malignancy.^12,13,14^ Small and slow-growing melanomas can be monitored, but surgery is indicated for iridociliary melanomas that shed pigment on surrounding tissues, have pronounced vascularity, and increase in thickness and size.^15,16^ Without the need for further imaging, a prediagnosis of melanoma was made and surgery was planned based on the clinical presentation and AS-OCT findings regarding the size, internal structure, and extension of the lesion. AS-OCT utilizing 1310 nm wavelength light for image acquisition has less tissue penetration than UBM, but provides higher image resolution. For this reason, it is more useful in the evaluation of superficial lesions located in the anterior aspect of the iris.

Most ophthalmology clinics use USB or AS-OCT more than UBM. However, the resolution of AS-OCT is reduced in large, densely pigmented lesions that extend to the ciliary body and cause shadowing.

In summary, AS-OCT is a noninvasive, convenient, high-resolution imaging modality which is useful in the preliminary diagnosis stage of iris or angle masses with no marked ciliary body involvement.

## Figures and Tables

**Figure 2A:**
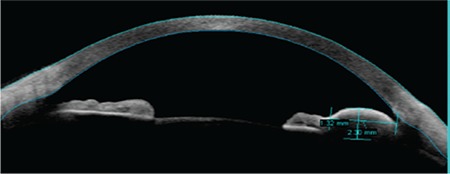
An iris mass measuring 2.30x1.32 mm extending to the angle is observed on anterior segment optical coherence tomography. The anterior aspect of the mass appears highly reflective (enhanced anterior segment single)

**Figure 2B:**
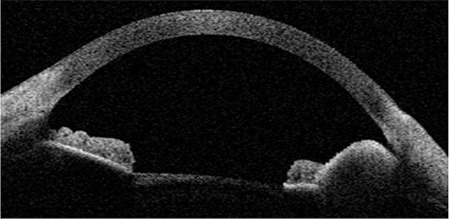
Shadowing is observed in anterior segment optical coherence tomography due to poor visibility of the posterior surface of iris (raw image mode)
